# Clinically relevant drug–drug interactions among elderly people with dementia

**DOI:** 10.1007/s00228-018-2514-5

**Published:** 2018-07-02

**Authors:** Eva Sönnerstam, Maria Sjölander, Hugo Lövheim, Maria Gustafsson

**Affiliations:** 1 Department of Pharmacology and Clinical Neuroscience, Division of Pharmacology, Umeå University, 901 87 Umeå, Sweden; 2 Department of Pharmacology and Clinical Neuroscience, Umeå University, 901 87 Umeå, Sweden; 3 Department of Community Medicine and Rehabilitation, Geriatric Medicine, Umeå University, 901 87 Umeå, Sweden

**Keywords:** Drug–drug interactions (DDIs), Drug-related problems (DRPs), Adverse drug reactions (ADRs), Adverse drug events (ADEs), Elderly people, Dementia

## Abstract

**Purpose:**

Increased numbers of drugs and changes in pharmacokinetic and pharmacodynamic parameters among elderly people contribute to increased prevalence of adverse drug reactions. Drug–drug interactions (DDIs) are an important reason for admission to hospital and elderly people with dementia are particularly vulnerable. The aims of the present study were to assess the occurrence and characteristics of clinically relevant DDIs and to investigate potential risk factors associated with DDIs among elderly people with dementia.

**Methods:**

People ≥ 65 years with dementia, admitted to two hospitals in Northern Sweden, were included. The medical records of 458 patients were reviewed. Clinically relevant DDIs were identified using the Janusmed interactions database. Pharmacological classification was conducted using Stockley’s classification system.

**Results:**

A total of 401 DDIs were identified among 43.2% of the study population, of which 98.5% had interactions that may require dose adjustment and 7.6% had drug combinations that should be avoided. Pharmacodynamic interactions were most common, of which furosemide–citalopram (*n* = 35) were most frequently observed. Omeprazol–citalopram (*n* = 25) was the most common drug combination among pharmacokinetic interactions. Citalopram and warfarin were the most commonly involved drug substances. An association was found between a higher number of medications being prescribed and having at least one DDI.

**Conclusion:**

Clinically relevant drug–drug interactions are prevalent among elderly people with dementia living in Northern Sweden. Drug–drug interactions should be identified in order to manage and prevent adverse outcomes. This is particularly important among this group of people especially when multiple medications are being prescribed.

## Introduction

Age-associated physiological changes result in pharmacokinetic and pharmacodynamic alterations in the body among elderly people. Pharmacodynamic changes usually increase the sensitivity to several drug classes, for example, psychotropic drugs [1]. This leads to altered drug effect, increased vulnerability to adverse drug reactions (ADRs) and drugs often exhibiting narrower therapeutic ranges [1, 2].

More pronounced changes in neurotransmitter substances, for example, acetylcholine and dopamine, have been observed in the central nervous system (CNS) among people with dementia than people without dementia [3], and an association has been found between lowered serotonin levels and Alzheimer’s disease [4]. Additionally, there are reports indicating changes in the blood–brain barrier among people with Alzheimer’s disease, which alter the possibility of drugs traversing the blood–brain barrier and reaching CNS. This may further alter drug response [5]. Thus, elderly people with dementia are extremely sensitive to ADRs [3].

Problems associated with drug treatment such as ADRs and potentially inappropriate drug use are common and are the reason for the high volume of hospitalisations among elderly people [6]. People with dementia or cognitive impairment are at even greater risk, in which 41% of hospital admissions were judged to be attributable, or partially attributable, to drug-related problems (DRPs) [7].

Drug–drug interactions (DDIs) defined as “when the effects of one drug are changed by the presence of another drug” [8] are a significant cause of these DRPs. For example, one review found that DDIs were responsible for 4.8% of admissions among elderly people [9]. Another study found that 17% of the ADRs causing hospitalisation were DDIs [10]. Many hospitalisations caused by DDIs could be avoided [11]. Increased risk of bleeding, prolongation of QT interval and hyperkalaemia are examples of the consequences of DDIs that expose elderly people with dementia to unnecessary risks if drug use is not properly monitored [12, 13].

Previous studies have found that inappropriate drugs according to age and inappropriate drugs according to renal function were prevalent in the study sample [14, 15]. It was also found that DDIs accounted for 6.9% of the drug-related hospitalisations in this population including elderly persons with dementia or cognitive impairment [7].

The objective of this study was therefore to further assess drug use among people aged 65 years or older with dementia and cognitive impairment, more specifically to assess the occurrence and character of all clinically relevant DDIs prevalent at admission to hospital. A further objective was to investigate factors associated with clinically relevant DDIs in old people with dementia or cognitive impairment. To the best of our knowledge, no previous studies have investigated this before.

## Methods

### Settings and study design

This cross-sectional study included the same persons involved in an intervention study [16]. In the intervention study, elderly people with dementia or cognitive impairment were recruited from two hospitals in Northern Sweden in order to investigate if the rate of readmissions was reduced when clinical pharmacists were included in the healthcare team. People from the acute internal medicine ward and orthopaedic ward at Umeå University Hospital and people admitted to the medicine wards at the county hospital in Skellefteå were recruited. People aged 65 years or older with dementia or cognitive impairment admitted to the included wards between 9 January 2012 and 2 December 2014 were asked to participate in the study. In total, 473 persons were invited and 13 declined to participate [16]. The present study population comprised both intervention and control groups at the time of index admission and before any intervention was performed. Persons with planned admissions (*n* = 1) and those who chose to withdraw from the intervention study before discharge (*n* = 1) were excluded, resulting in a final study sample of 458 persons.

### Data extraction and definitions

The medications used by persons at admission (before any intervention was performed) to the included hospital wards were collected from the relevant person’s medical records. Number of medications, drug formulation and administration, age, sex, type of dementia, Mini Mental State Examination (MMSE) score when available, type of accommodation and geographic location were also collected from the relevant person’s medical records. Prescriptions with pro re nata dose were not included in the analysis (due to uncertainty about the dose) nor were over-the-counter drugs (due to lack of information in the medical records).

#### Definitions

Clinically relevant DDIs were identified using the Janusmed interaction database (formerly called Sfinx) [17, 18] in accordance with a previous study [13]. This is a computerised system listing information about DDIs based on pairwise combination of drug substances. Information about interactions is updated 4–10 times per year. Drug interactions are classified into four different categories, A–D, dependent on clinical relevance, in which category C and D are considered to be clinically relevant DDIs (Table 1). Moreover, the extent of documentation is recorded as 0–4 relating to the level of documentation (Table 1) [18].

**Table 1:** Classification of drug–drug interactions in Sweden, their clinical relevance and level of documentation [18]

Clinical relevance	
A: Clinically insignificant DDIs.	
B: Clinical relevance is unknown and/or varies.	
C: Clinically relevant DDIs that can be handled with individual dose adjustment, for example.	
D: Clinically relevant DDIs that should be avoided.	
Level of documentation	
0: Data from studies including other drug substances with similar properties.	
1: Data from incomplete case reports and/or in vitro studies.	
2: Data from well-documented case reports.	
3: Data from studies on healthy individuals and/or pilot studies on patients.	
4: Data from controlled studies on relevant study population.	

*DDIs* drug–drug interactions

The interactions selected for this study focused on potentially clinically relevant DDIs type C, which may require dose adjustment, or type D, which should be avoided, consistent with previous studies [19–22] regardless of the level of documentation [19]. DDIs were identified on drug substance level, meaning that drug formulations with several drug substances may give rise to several signals in combination with another drug substance. Identified DDIs were further categorised regarding pharmacological mechanisms according to Stockley’s classification system (Table 2) [8]. The categorisation was based on the information given in the Janusmed interaction database and Stockley’s Interaction Checker [17, 23]. Interactions with uncertainty of classification were categorised as “classification uncertain”.

**Table 2:** Categorisation of pharmacological mechanism according to Stockley’s classification system [8]

1. Pharmacokinetic interactions:	
1.1 Drug absorption interactions.	
1.2 Drug distribution interactions.	
1.3 Drug metabolism (biotransformation) interactions.	
1.3.1 Enzyme induction	
1.3.2 Enzyme inhibition	
1.4 Drug excretion interactions.	
1.5 Drug transporter proteins.	
2. Pharmacodynamic interactions:	
2.1 Additive or synergistic interactions.	
2.2 Antagonistic or opposing interactions.	
2.3 Drug or neurotransmitter uptake interactions.	

### Data analysis

Descriptive statistics were used to present the characteristics of the study sample. Frequencies were calculated for dichotomous variables, i.e. sex, type of accommodation (living at home or in a nursing home) and geographic location (Skellefteå or Umeå). Continuous variables, i.e. age, number of medications at admission and MMSE score, were presented as mean values with standard deviation (SD).

Simple logistic regression analyses were conducted to investigate the association between the potential risk factors collected from the medical records, i.e. sex, age, number of medications at admission, MMSE score, type of accommodation and geographic location, and having at least one clinically relevant DDI. Multiple logistic regression analysis was conducted to investigate the association between sex, age and significant variables from the simple model, and having at least one potentially clinically relevant DDI. The results are presented as odds ratios (ORs) with 95% confidence intervals (CIs). All analyses were conducted using IBM SPSS Statistics 24.

## Results

Of 458 people in the study sample, 198 (43.2%) had at least one clinically relevant DDI. Among people with interactions, 195/198 (98.5%) had one or more DDI that required dose adjustment and 15/198 (7.6%) had at least one DDI that should be avoided. Among the study sample, 286 (62.4%) were women, the mean age was 83.2 ± 6.6, mean number of medications at admission was 7.7 ± 3.5 and mean MMSE score was 19.8 ± 4.7. A total of 307 (67.0%) of the study participants were living at home and 338 (73.8%) lived in Umeå municipality. An association was found in both the univariate and the multivariable model between having a higher number of medications prescribed and having at least one clinically relevant DDI. However, no associations were found between age, sex, MMSE score, type of accommodation or geographic location and the risk of having one or more clinically relevant DDIs in the logistic regression model(s) (Table 3).

**Table 3:** Descriptive statistics of study population and comparison between people with and without clinically relevant DDIs

Characteristics of study sample	Total	DDI(s)	No DDI	Simple OR (95% CI)	Multiple OR (95% CI)
Cases, *n*	458	198	260		
Sex, *n* (%)					
Female	286 (62.4)	121 (61.1)	165 (63.5)	0.905 (0.618–1.325)	0.853 (0.558–1.303)
Male	172 (37.6)	77 (38.9)	95 (36.5)	Ref.	
Age (years), mean ± SD, (range)	83.2 ± 6.6 (65–99)	83.2 ± 6.3	83.3 ± 6.8	0.998 (0.970–1.026)	1.008 (0.977–1.040)
Number of medications at admission, mean ± SD, (range)	7.7 ± 3.5 (0–20)	9.4 ± 3.3	6.5 ± 3.2	1.311 (1.227–1.402)	1.312 (1.227–1.403)
MMSE^a^ (0–30), mean ± SD, (range)	19.8 ± 4.7 (7–29)	19.5 ± 4.7	20.0 ± 4.7	0.979 (0.916–1.047)	–
Type of accommodation, *n* (%)					
Nursing home	151 (33.0)	62 (31.3)	89 (34.2)	0.876 (0.590–1.300)	–
Living at home	307 (67.0)	136 (68.7)	171 (65.8)	Ref.	
Geographic location, *n* (%)					
Skellefteå	120 (26.2)	45 (22.7)	75 (28.8)	0.725 (0.473–1.112)	–
Umeå	338 (73.8)	153 (77.3)	185 (71.2)	Ref.	

*CI* confidence interval, *DDI(s)* drug–drug interaction(s), *MMSE* Mini-Mental State Examination, *OR* odds ratio, *Ref* reference category, *SD* standard deviation

The multiple analysis includes sex, age and significant variables from the simple model (number of medications at admission)

^a^*n* = 162 because the test was not performed on every patient

A total of 401 clinically relevant DDIs were identified among the study sample of which 385 (96.0%) required dose adjustment and 16 (4.0%) cases involved drug combinations that should be avoided. Pharmacodynamic interactions were the most common type of identified interactions—187/401 (46.6%). Pharmacokinetic interactions accounted for 169/401 (42.1%) of the identified DDIs. Additive and synergistic interactions, 185/187 (98.9%), accounted for the vast majority of pharmacodynamic interactions. Furosemide–citalopram, 35/187 (18.7%), and acetylsalicylic acid–citalopram, 32/187 (17.1%), were the most common drug combinations found among pharmacodynamic interactions. Drug absorption interactions were the largest subclass, 83/169 (49.1%) among pharmacokinetic interactions. Omeprazole–citalopram, 25/169 (14.8%), and calcium–levothyroxine, 22/169 (13.0%), were the most common drug combinations among pharmacokinetic DDIs. Increased risk of bleeding and reduced efficacy were the most common potential clinical consequences of the identified DDIs (Table 4).

**Table 4:** Frequency of clinically relevant DDIs, their pharmacological and clinical classification and potential consequence

Classification pharmacological	Classification clinical/documentation	Drug combination (frequency)	Potential clinical consequence
1. Pharmacokinetic interactions (*n* = 169)			
1.1 Drug absorption interactions (*n* = 83)	C3	Calcium + alendronic acid (13)	Reduction of efficacy of alendronic acid
	C3	Calcium + levothyroxine (22)	Reduction of efficacy of levothyroxine
	C3	Calcium + iron (15)	Reduction of efficacy of iron
	C0	Calcium + risedronic acid (2)	Reduction of efficacy of risedronic acid
	D3	Colestyramine + furosemide (1)	Reduction of efficacy of furosemide
	C4	Iron + levothyroxine (8)	Reduction of efficacy of levothyroxine
	C3	Iron + levodopa (2)	Reduction of efficacy of levodopa
	C0	Iron + alendronic acid (2)	Reduction of efficacy of alendronic acid
	C0	Iron + risedronic acid (1)	Reduction of efficacy of risedronic acid
	C3	Iron + aluminium (1)	Reduction of efficacy of iron
	C3	Iron + sodium hydrogen carbonate (13)	Reduction of efficacy of iron
	C3	Magnesium + mycophenolic acid (1)	Reduction of efficacy of mycophenolic acid
	C1	Magnesium + calcium polystyrene sulphonate (1)	Increased risk of systemic metabolic alkalosis
	C3	Omeprazole + levothyroxine (1)	Reduction of efficacy of levothyroxine
1.2.1 Drug metabolism interactions, enzyme induction (*n* = 15)	C2	Azathioprine + warfarin (1)	Reduction in clinical efficacy of warfarin
	C3	Carbamazepine + acetaminophen (1)	Increased risk of liver damage
	C1	Carbamazepine + atorvastatin (1)	Reduction in clinical efficacy of atorvastatin
	C2	Carbamazepine + cholecalciferol (2)	Reduction in clinical efficacy of cholecalciferol
	C4	Carbamazepine + citalopram (2)	Reduction in clinical efficacy of citalopram
	D0	Carbamazepine + diazepam (1)	Reduction in clinical efficacy of diazepam
	D3	Carbamazepine + felodipine (1)	Reduction in clinical efficacy of felodipine
	C4	Carbamazepine + olanzapine (1)	Reduction in clinical efficacy of olanzapine
	D0	Carbamazepine + ticagrelor (1)	Reduction in clinical efficacy of ticagrelor
	C3	Phenytoin + acetaminophen (1)	Reduction in clinical efficacy of acetaminophen, increased risk of liver damage
	C3	Phenytoin + mirtazapine (1)	Reduction in clinical efficacy of mirtazapine
	C1	Phenytoin + simvastatin (1)	Reduction in clinical efficacy of simvastatin
	C3	Prednisolone + tacrolimus (1)	Reduction in clinical efficacy of tacrolimus
1.2.2 Drug metabolism interactions, enzyme inhibition (*n* = 66)	C3	Amiodarone + simvastatin (1)	Increased efficacy of simvastatin, risk of myopathy
	C4	Amiodarone + metoprolol (2)	Increased risk of hypotension, bradycardia or cardiac arrest
	C1	Atorvastatin + diltiazem (1)	Increased efficacy of atorvastatin, risk of myopathy
	D2	Budesonide + fluconazole (1)	Increased plasma levels of budesonide
	C4	Ciclosporin + felodipine (1)	Increased plasma levels of felodipine
	C3	Ciclosporin + cinacalcet (1)	Increased efficacy of cinacalcet
	C0	Cinacalcet + metoprolol (1)	Increased efficacy of metoprolol
	C0	Clopidogrel + imatinib (1)	Increased efficacy of imatinib
	C3	Darunavir + atorvastatin (1)	Increased efficacy of atorvastatin, risk of myopathy
	C0	Darunavir + budesonide (1)	Increased efficacy of budesonide
	D1	Econazole + warfarin (1)	Increased efficacy of warfarin
	C3	Esomeprazole + clopidogrel (3)	Reduction in clinical efficacy of clopidogrel
	C0	Esomeprazole + escitalopram (1)	Increased plasma levels of escitalopram, risk of QT prolongation
	C0	Esomeprazole + citalopram (2)	Increased plasma levels of citalopram, risk of QT prolongation
	C0	Fluconazole + clopidogrel (1)	Reduction in clinical efficacy of clopidogrel
	D2	Fluconazole + citalopram (1)	Increased plasma levels of citalopram, risk of QT prolongation
	C3	Hydroxychloroquine + metoprolol (1)	Increased efficacy of metoprolol
	C0	Imatinib + atorvastatin (1)	Increased efficacy of atorvastatin, risk of myopathy
	C3	Mirabegron + metoprolol (2)	Increased efficacy of metoprolol, risk of hypotension and bradycardia
	C3	Omeprazole + citalopram (25)	Increased plasma levels of citalopram, risk of QT- prolongation
	C3	Omeprazole + clopidogrel (7)	Reduction in clinical efficacy of clopidogrel
	C3	Omeprazole + escitalopram (4)	Increased plasma levels of escitalopram, risk of QT-prolongation
	C2	Omeprazole + tacrolimus (1)	Increased efficacy of tacrolimus
	C0	Paroxetine + timolol (1)	Increased efficacy of timolol
	C3	Phenytoin + losartan (1)	Reduction in clinical efficacy of losartan
	C4	Ritonavir + atorvastatin (1)	Increased efficacy of atorvastatin, risk of myopathy
	C2	Ritonavir + budesonide (1)	Increased efficacy of budesonide
	D4	Warfarin + sulfamethoxazole^a^ (1)	Increased risk of bleeding
1.3 Drug excretion interactions (*n* = 5)	C3	Spironolactone + digoxin (4)	Increased plasma levels of digoxin, risk of digoxin toxicity
	C1	Furosemide + lithium (1)	Decreased urinary excretion of lithium, risk of lithium toxicity
2. Pharmacodynamic interactions (*n* = 187)			
2.1 Additive or synergistic interactions (*n* = 185)	C0	Acetylsalicylic acid + venlafaxine (2)	Increased risk of bleeding
	C4	Acetylsalicylic acid + sertraline (9)	Increased risk of bleeding
	C4	Acetylsalicylic acid + escitalopram (2)	Increased risk of bleeding
	C4	Acetylsalicylic acid + citalopram (32)	Increased risk of bleeding
	C3	Acetylsalicylic acid + ibuprofen (1)	Increased risk of bleeding, reduced cardioprotective efficacy
	C0	Clopidogrel + citalopram (8)	Increased risk of bleeding
	C0	Clopidogrel + escitalopram (2)	Increased risk of bleeding
	C0	Clopidogrel + paroxetine (1)	Increased risk of bleeding
	C0	Clopidogrel + sertraline (6)	Increased risk of bleeding
	C0	Clopidogrel + tramadol (1)	Increased risk of bleeding
	C0	Clopidogrel + venlafaxine (1)	Increased risk of bleeding
	C0	Dalteparin + escitalopram (1)	Increased risk of bleeding
	C0	Dalteparin + citalopram (1)	Increased risk of bleeding
	C0	Dalteparin + sertraline (1)	Increased risk of bleeding
	C0	Dipyridamole + citalopram (3)	Increased risk of bleeding
	C4	Ibuprofen + citalopram (1)	Increased risk of bleeding
	C0	Fondaparinux + citalopram (1)	Increased risk of bleeding
	D4	Warfarin + diclofenac (1)	Increased risk of bleeding
	C3	Warfarin + levothyroxine (10)	Increased risk of bleeding
	C4	Warfarin + citalopram (11)	Increased risk of bleeding
	C1	Warfarin + prednisolone (7)	Increased risk of bleeding
	C2	Warfarin + sertraline (1)	Increased risk of bleeding
	C2	Bendroflumethiazide + citalopram (3)	Increased risk of hyponatraemia
	C2	Furosemide + escitalopram (1)	Increased risk of hyponatremia
	C2	Furosemide + citalopram (35)	Increased risk of hyponatraemia
	C2	Furosemide + sertraline (9)	Increased risk of hyponatraemia
	C3	Hydrochlorothiazide + citalopram (2)	Increased risk of hyponatraemia
	C4	Amiloride + enalapril (1)	Increased risk of hyperkalaemia
	C4	Spironolactone + candesartan (1)	Increased risk of hyperkalaemia
	C4	Spironolactone + enalapril (11)	Increased risk of hyperkalaemia
	C4	Spironolactone + losartan (7)	Increased risk of hyperkalaemia
	C3	Spironolactone + potassium (1)	Increased risk of hyperkalaemia
	C4	Spironolactone + ramipril (2)	Increased risk of hyperkalaemia
	D0	Ciprofloxacin + escitalopram (1)	Increased risk of QT- prolongation
	D0	Ciprofloxacin + citalopram (1)	Increased risk of QT- prolongation
	D0	Citalopram + donepezil (2)	Increased risk of QT- prolongation
	D1	Citalopram + haloperidol (1)	Increased risk of QT- prolongation
	D0	Diltiazem + timolol (1)	Increased risk of hypotension, bradycardia or cardiac arrest
	D0	Verapamil + timolol (1)	Increased risk of hypotension, bradycardia or cardiac arrest
	C3	Methotrexate + sulfamethoxazole^a^ (1)	Additive, antagonistic effect on folic acid synthesis
	C3	Methotrexate + trimethoprim^a^ (1)	Additive, antagonistic effect on folic acid synthesis
2.2 Antagonistic or opposing interactions (*n* = 2)	C4	Furosemide + diclofenac (1)	Decreased effect of loop diuretics leading to decreased diuresis and worsening of heart failure
	C0	Diclofenac + carvedilol (1)	Decreased effect of beta-blocking agents
3. Classification uncertain (*n* = 45)			
	C4	Ciclosporin + pravastatin (1)	Increased efficacy of pravastatin, risk of myopathy
	C4	Warfarin + acetaminophen (26)	Increased risk of bleeding
	C3	Morphine + gabapentin (1)	Increased risk of CNS-symptoms
	C0	Ramipril + darbepoetin alfa (1)	Decreased responsiveness to darbepoetin alfa
	C4	Warfarin + simvastatin (16)	Increased efficacy of warfarin, risk of bleeding

C = interaction which may require dose adjustment

D = interaction which should be avoided

0–4 = level of documentation according to Table 1

^a^Combined formulation: trimethoprim/sulfamethoxazole

Citalopram and warfarin were the most commonly involved drug substances among the clinically relevant DDIs in both C and D interactions (Table 5).

**Table 5:** Identified drug substances and their frequency of involvement in clinically relevant DDIs, category C and D

Drug	DDI(s) category C, *n* (%)^a^	DDI(s) category D, *n* (%)^b^
Acetaminophen	28 (7.3)	
Acetylsalicylic acid	46 (11.9)	
Alendronic acid	15 (3.9)	
Aluminium	1 (0.3)	
Amiloride	1 (0.3)	
Amiodarone	3 (0.8)	
Atorvastatin	5 (1.3)	
Azathioprine	1 (0.3)	
Bendroflumethiazide	3 (0.8)	
Budesonide	2 (0.5)	1 (6.3)
Calcium	52 (13.5)	
Calcium polystyrene sulphonate	1 (0.3)	
Candesartan	1 (0.3)	
Carbamazepine	7 (1.8)	3 (18.8)
Carvedilol	1 (0.3)	
Cholecalciferol	2 (0.5)	
Ciclosporin	3 (0.8)	
Cinacalcet	2 (0.5)	
Ciprofloxacin		2 (12.5)
Citalopram	126 (32.7)	5 (31.3)
Clopidogrel	31 (8.1)	
Colestyramine		1 (6.3)
Dalteparin	3 (0.8)	
Darbepoetin alfa	1 (0.3)	
Darunavir	2 (0.5)	
Diazepam		1 (6.3)
Diclofenac	2 (0.5)	1 (6.3)
Digoxin	4 (1.0)	
Diltiazem	1 (0.3)	1 (6.3)
Dipyridamole	3 (0.8)	
Donepezil		2 (12.5)
Econazole		1 (6.3)
Enalapril	12 (3.1)	
Escitalopram	11 (2.9)	1 (6.3)
Esomeprazole	6 (1.6)	
Felodipine	1 (0.3)	1 (6.3)
Fluconazole	1 (0.3)	2 (12.5)
Fondaparinux	1 (0.3)	
Furosemide	47 (12.2)	1 (6.3)
Gabapentin	1 (0.3)	
Haloperidol		1 (6.3)
Hydrochlorothiazide	2 (0.5)	
Hydroxychloroquine	1 (0.3)	
Ibuprofen	2 (0.5)	
Imatinib	2 (0.5)	
Iron	42 (10.9)	
Levodopa	2 (0.5)	
Levothyroxine	41 (10.6)	
Lithium	1 (0.3)	
Losartan	8 (2.1)	
Magnesium	2 (0.5)	
Methotrexate	2 (0.5)	
Metoprolol	6 (1.6)	
Mirabegron	2 (0.5)	
Mirtazapine	1 (0.3)	
Morphine	1 (0.3)	
Mycophenolic acid	1 (0.3)	
Olanzapine	1 (0.3)	
Omeprazole	38 (9.9)	
Paroxetine	2 (0.5)	
Phenytoin	4 (6.2)	
Potassium	1 (0.3)	
Pravastatin	1 (0.3)	
Prednisolone	8 (2.1)	
Ramipril	3 (0.8)	
Risedronic acid	3 (0.8)	
Ritonavir	2 (0.5)	
Sertraline	26 (6.8)	
Simvastatin	18 (4.7)	
Sodium hydrogen carbonate	13 (3.4)	
Spironolactone	26 (6.8)	
Sulfamethoxazole	1 (0.3)	1 (6.3)
Tacrolimus	2 (0.5)	
Ticagrelor		1 (6.3)
Timolol	1 (0.3)	2 (12.5)
Tramadol	1 (0.3)	
Trimethoprim	1 (0.3)	
Venlafaxine	3 (0.8)	
Verapamil		1 (6.3)
Warfarin	72 (18.7)	3 (18.8)

*C* interaction, which may require dose adjustment, *D* interaction, which should be avoided, *DDI(s)* drug–drug interaction(s)

^a^Based on the total number of clinically relevant DDIs, category C, *n* = 385

^b^Based on the total number of clinically relevant DDIs, category D, *n* = 16

## Discussion

In the current study, almost half of the individuals with dementia or cognitive impairment admitted to hospital had at least one potentially clinically relevant DDI, which can be regarded as a high proportion being at risk of experiencing adverse drug reactions. The prevalence of people with potentially clinically relevant DDIs in the present study is, however, in the middle range compared to other studies [12, 20–22]. Different study designs, settings and inclusion criteria might explain the different prevalence of DDIs [24, 25]. Another explanation for the difference might be the extensive work on medication reviews in the county, which lower potentially clinically relevant DDIs among the study sample [26]. The prevalence is, however, still high and indicates an opportunity for further improvement.

The average number of drugs at the time of admission was higher among participants in the present study compared to other studies [12, 22, 25]. It is known that elderly people, and especially those with dementia, take on average more medications than people without dementia [27], which may be due to multiple comorbidities, experienced symptoms and misinterpretation of adverse drug reactions, resulting in new prescribed medications [24]. This might explain the high average of drug use compared to other studies, a risk factor that has been found to have associations with the presence and increased risk of having DDIs [12, 24, 25, 28, 29], which was also found in the present study. No association was found in the present study between sex and the presence of DDI. This result is consistent with other studies stating the same result [13, 29]. Conflicting results were, however, found in another study which stated that DDIs are more common in men than in women [19]. No association was found in the present study between age and having one or more DDI. Another study found that a younger age was associated with the presence of DDIs [13]. Different study settings may explain the different results. No association was found between MMSE score and having DDIs, which is consistent with previous results [25]. Nor were any associations found in the present study between living accommodation and geographic location, and the presence of one or more DDIs.

Most of the interactions in the present study required dose adjustment or some monitoring in order to prevent adverse drug reactions (C interactions). Furosemide–citalopram and acetylsalicylic acid–citalopram were the most common DDIs among pharmacodynamic interactions. If not monitored correctly, their additive or synergistic interaction may lead to hyponatraemia and increased risk of bleeding, respectively [17]. Omeprazole–citalopram and calcium–levothyroxine were the most common pharmacokinetic interactions leading to prolongation of QT interval due to enzyme inhibition and reduced efficacy due to absorption interactions. Warfarin–acetaminophen were also among the most common DDIs found in the present study leading to increased risk of bleeding if no dose adjustments were made [17]. The C interactions found in the present study differ both in prevalence and from the most common DDIs identified in other studies, in which citalopram–antiplatelet, non-steroidal anti-inflammatory drugs (NSAIDs)–beta-blocking agents, angiotensin-converting enzyme (ACE) inhibitors–NSAIDs and digoxin–furosemide were the most commonly reported interactions [12, 20–22, 29]. The low prevalence of interactions involving NSAIDs in the present study might be attributable to information campaigns to physicians and medication reviews performed on people living in nursing homes in the county of Västerbotten [30].

Even if a small proportion of the study population had interactions that should be avoided (D interactions), it warrants concern because of the difficulty in managing these interactions compared to C interactions [31]. Citalopram–donepezil was the most common D interaction in the present study and this differs from the most common D interactions found in other studies [13, 19, 20, 29]. The reason for the difference is the specific study population in the present study. This severe interaction is therefore of concern specifically among people with dementia. Citalopram is commonly used to treat depression among elderly people with dementia in Sweden and the drug can lead to a prolongation of the QT interval that is not seen among all serotonin selective reuptake inhibitors (SSRI). Together with donepezil, an additive prolongation effect on QT interval may lead to torsades de pointes, a serious side effect [17]. Further, it is recommended that SSRI is used instead of antipsychotic drugs to treat behavioural and psychological symptoms in dementia (BPSD) when non-pharmacological interventions or treatment with memantine do not work effectively [32]. Citalopram was the most commonly involved drug substance among the identified DDIs in the present study. This is consistent with a study which found that psychotropic drug use was commonly involved in DDIs among elderly people with dementia [22]. Another study found an association between depression and having one or more DDIs [12]. It is therefore important to regularly evaluate the need for antidepressants and monitor the side effects, e.g. increased risk of bleeding and hyponatraemia due to potential DDIs, which may arise with other concomitant drugs, especially among elderly people with dementia [32, 33]. Warfarin was the second most commonly involved drug among the DDIs in the present study. Its increased anticoagulant effect among elderly people, significant dose-response variability, narrow therapeutic index and the difficulties with compliance that are sometimes experienced among people with dementia make DDIs involving warfarin additionally complex and important to monitor to avoid increased risk of bleeding [1, 34, 35]. Warfarin treatment is usually well monitored, which fortunately reduces the risks of adverse outcomes [36].

The most common clinical consequences were increased risk of bleeding, reduced efficacy of involved drugs and electrolyte disturbances, which may have been caused by the identified interactions in the present study. Some of the identified interactions also led to hospitalisation, which was mentioned in another study investigating drug-related hospital admissions [7] among the same study sample as in the present study. In that study, it was found that 6.9% of hospital admissions were due to drug–drug interactions [7]. Increased risk of bleeding is of special concern among elderly people [35] and particularly among those with dementia because the clinical presentation may be atypical and involve confusion. The adverse outcome is then particularly difficult to identify [34]. Some cases may be monitored via laboratory tests, dose adjustments or additional drug therapy, e.g. proton pump inhibitors (PPIs) to prevent bleeding. This may, however, increase the risk of DDIs depending on the drug chosen. Electrolyte disturbances are important to identify and manage particularly when new drug treatments are introduced and among elderly women who are more vulnerable to hyponatraemia, for example [17]. Increased risk of bleeding and electrolyte disturbances were also the most common reasons for hospital admission due to DDIs among the present study sample [7]. ADRs and reduced efficacy may be identified and managed among those living in nursing homes but might result in reduced quality of life among people living at home, as they are unable to identify and communicate adverse outcomes by themselves.

The following limitations should be recognised. Drugs with pro re nata dosage were excluded, which may lower the prevalence of clinically relevant DDIs in the present study. Even if the DDIs were categorised as clinically relevant, the prevalence of interactions may not reflect their true clinical relevance. We did not know if doses had been correctly adjusted, e.g. warfarin–acetaminophen, if drugs were taken separately when this was recommended, e.g. calcium–levothyroxine, or if the drug combination was appropriate according to the clinical indication, e.g. spironolactone–enalapril in individuals with congestive heart failure [17]. Nor was it possible to evaluate causality or outcome due to the cross-sectional study design. It was only possible to check interactions pairwise [18]. Interactions from more than two substances have therefore not been identified. Moreover, only some specific pharmacodynamic interactions have been included in the database [18].

The strength of the present study is that the result can be considered as representative for people 65 years or older with dementia because no other inclusion or exclusion criteria were applied. Out of 473 invited people, only two were excluded and only 13 declined to participate. Moreover, medical records are a reliable source of information in a cross-sectional study [37]. The Janusmed interaction database is a regularly updated DDI identification tool used in clinical practice in Sweden [18].

## Conclusion

Clinically relevant DDIs are prevalent among elderly people with dementia in Northern Sweden and interactions requiring dose adjustment are most common. Thus, identification and clinical and laboratory assessments of DDIs are crucial in order to manage and prevent adverse outcomes among elderly people with dementia, especially when multiple medications are being prescribed.
